# A Critical Appraisal of the Chronic Pain Rate After Inguinal Hernia Repair

**DOI:** 10.3389/jaws.2023.10972

**Published:** 2023-01-19

**Authors:** Anders Gram-Hanssen, Stina Öberg, Jacob Rosenberg

**Affiliations:** Center for Perioperative Optimization, Herlev Hospital, Copenhagen University Hospital, Herlev, Denmark

**Keywords:** chronic pain, quality of life, inguinal hernia, review of literature, critical appraisal

## Abstract

**Purpose:** To critically appraise highly cited studies reporting on the rate of chronic pain after inguinal hernia repair.

**Methods:** Google Scholar was searched on 23 May 2022. We only included publications with more than 10 citations per year since publication and more than 100 citations in total. Both reports of original data and systematic reviews were included. Risk of bias and quality of the included studies were assessed with either the Joanna Briggs Institute Checklist for Prevalence Studies or the AMSTAR 2 depending on study design.

**Results:** Twenty studies were included and evaluated. The rate of chronic postoperative inguinal pain of any degree ranged from 10%–63%, and the rate of moderate-to-severe pain ranged from 1%–18%. All studies reported the rate of pain of any degree, and most studies reported the rate of moderate-to-severe pain influencing daily activities. Studies used different temporal definitions of chronic pain, but most studies defined it as pain persisting either three or six months postoperatively. Ten studies used unvalidated questionnaires or significantly modified versions of validated questionnaires. Eleven studies primarily included patients receiving open repair. Included studies had median 21 citations per year (range 10–39) and median 387 citations in total (range 127–788).

**Conclusion:** The rates of chronic postoperative inguinal pain reported in the included highly cited studies are possibly inaccurate, excessive, and outdated. New prospective studies based on uniform definitions and standards of measurement are warranted to better assess a contemporary chronic pain rate after inguinal hernia repair.

## Introduction

Chronic postoperative inguinal pain is arguably the most important and patient-centered outcome of inguinal hernia repair [1]. Chronic pain is a dreaded long-term complication for patients and likely more so than recurrences and reoperations. However, it is still an area of research that is inadequately understood [2–4], and chronic pain continues to present complicated diagnostic and therapeutic challenges [5–7].

The exact extent of the problem—i.e., the rate of chronic pain after inguinal hernia repair—is unclear. The rates conventionally reported in the literature vary considerably, and some sources report rates from 0% to 37% [8]. This large variation can likely be explained by several factors: studies use different definitions of chronic pain, different means of measurement, and different times of follow-up [4, 8, 9]. In addition, the leading studies in the field are older and possibly outdated, and these highly cited studies may not adequately reflect the ongoing advances in surgical practice in recent years, and a significant decrease in the occurrence of chronic pain may be expected. The recent advances in hernia surgery include an increased specialisation and development of certified hernia centres, more focus on training and recognition of specialist hernia surgeons [10, 11], advances in surgical device development including mesh technology, and a growing scientific focus on hernia research [12]. However, despite these developments, older and likely outdated chronic pain rates are still frequently repeated in the literature and are also communicated to patients preoperatively.

We hypothesise that the rates of chronic pain after inguinal hernia repair conventionally reported in the literature are outdated and exaggerated. In this review, we wanted to substantiate this claim through a critical appraisal of highly cited studies in the field and provide a discussion of its implications.

## Methods

In this review, we only included highly cited studies reporting on the chronic pain rate after inguinal hernia repair. The included studies were identified through the literature search engine Google Scholar [13], and the search was performed on 23 May 2022 using the search terms “hernia,” “groin,” “pain,” and “herniorrhaphy.” The applied inclusion criteria were: studies with original data or systematic reviews with extractable data on the chronic pain rate, >10 citations per year since publication in Google Scholar, and >100 citations overall.

From the included studies, we extracted general study information, bibliometric data, reported rates of chronic pain, reported severity of chronic pain, and the applied temporal definition of chronic pain (i.e., timepoint where study authors considered pain to have become chronic). If a study reported on multiple follow-ups, we extracted data from the shortest follow-up where pain was defined as chronic.

We performed a risk of bias assessment of the included original studies using the Joanna Briggs Institute Checklist for Prevalence Studies (JBI) [14]. The JBI is a critical appraisal tool that can be applied independently of study design. It was developed specifically for evaluating the validity of prevalence data, and it does not consider the methodological quality of studies in other regards. For the specifics on using and interpreting the JBI, we refer to the literature [14]. We evaluated the included systematic reviews using the AMSTAR 2 checklist [15].

## Results

**FIGURE 1:**
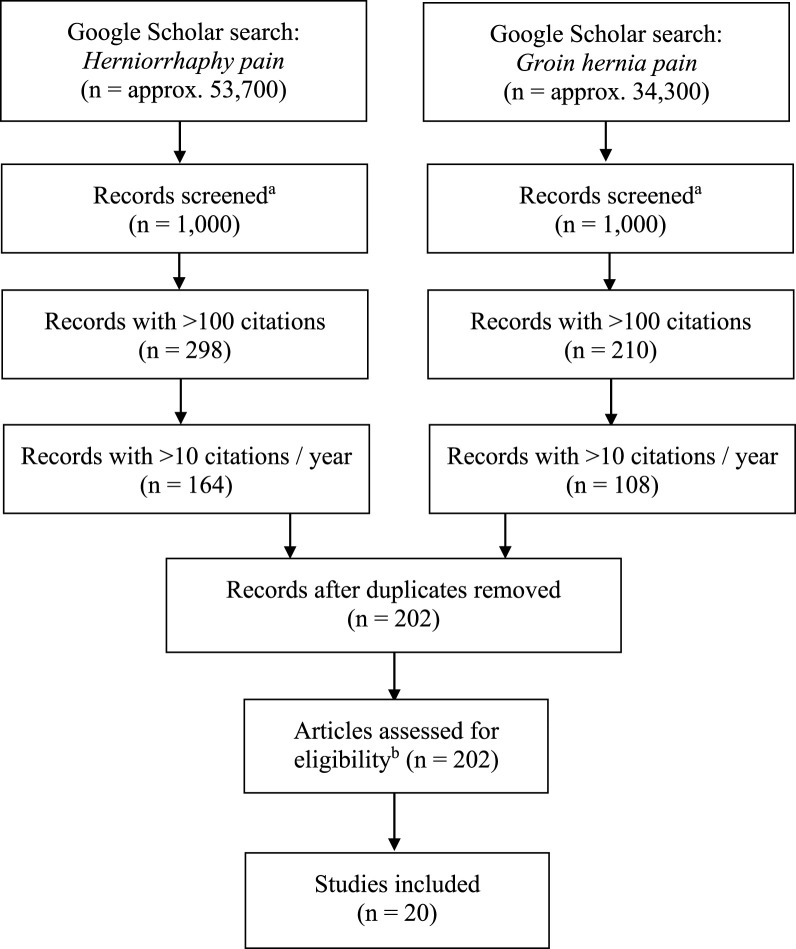
Literature search flow-chart. a: The Google Scholar search engine only allows for viewing of 1,000 records per search query. b: According to the remaining inclusion criteria. Approx., approximately.

In total, 20 publications fulfilled the inclusion criteria and were selected for critical appraisal [16–35] (Table 1; Figure 1). These included three systematic reviews [23, 27, 31], six randomised clinical trials [16, 18, 25, 26, 28, 34], and 11 observational studies [17, 19, 20, 21, 22, 24, 29, 30, 32, 33, 35]. Nine of the randomised clinical trials and observational studies [16–22, 28, 29] were also included in at least one of the three included systematic reviews [23, 27, 31]. Therefore, we have not conducted a traditional meta-analysis, and instead we have only provided summarised ranges. The reported chronic pain rates in the selected publications ranged widely, and many different definitions of chronic pain were applied. The included studies used different methods of measurement, of which the most frequent were ad-hoc (non-standardised) questionnaires. Studies had median 21 citations per year (range 10–39) and median 387 citations in total (range 127–788). The included studies were published between 1996 and 2011, and all of them remain frequently cited to this day (Table 1).

**TABLE 1:** Highly cited publications on chronic postoperative inguinal pain.

First author [ref.]	Year of publication	Study design^a^	Citation count^b^		Surgical approach		Temporal definition of chronic pain (months)	Chronic pain rate (%)		Measurement instrument
			Overall	Per year	Lap/Open	Mesh/non-mesh		Any pain	Significant pain^c^	
Cunningham [16]	1996	RCT	532	20	Open	Non-mesh	12	63	12	VRS
Callesen [17]	1999	Observational	518	23	Open	Both	12^d^	19	6	Ad-hoc
MRC LGH Trial Group [18]	1999	RCT	356	15	Both	Both	12^d^	32^e^	-	Ad-hoc
Bay-Nielsen [19]	2001	Observational	788	38	Both	Both	12^d^	29	11	Ad-hoc
Poobalan [20]	2001	Observational	577	27	Open	Both	3	30	-	Ad-hoc, MPQ, SF-36, UCSF^f^
Courtney [21]	2002	Observational	359	18	Both	Both	3^g^	46	3^g^	Ad-hoc, SF-36^g^
Kumar [22]	2002	Observational	365	18	Both	Mesh	-^h^	30^h^	18^h^	Ad-hoc
Poobalan [23]	2003	Review	715	38	Both	Both	3^i^	54^i^	-	-
Bay-Nielsen [24]	2004	Observational	271	15	Open	Both	6^j^	23	-	Ad-hoc
Grant [25]	2004	RCT	217	12	Both	Both	-^k^	32^k^	3^k^	Ad-hoc
Köninger [26]	2004	RCT	191	11	Both	Both	-^l^	29^l^	9^l^	Interview, VAS
Aasvang [27]	2005	Review	664	39	Both	Both	6^m^	12	-	-
O’Dwyer [28]	2005	RCT	448	26	Open	Mesh	12^n^	42^n^	3^n^	Ad-hoc, SF-36, VAS^n^
Alfieri [29]	2006	Observational	227	14	Open	Mesh	6^o^	10	2	VRS
Fränneby [30]	2006	Observational	436	27	Both	Both	-^p^	31	6	DIBS
Nienhuijs [31]	2007	Review	453	30	Both	Mesh	3^q^	11	-^q^	-
Kalliomäki [32]	2008	Observational	146	10	Both	Both	-^r^	30	6	IPQ
Aasvang [33]	2010	Observational	409	34	Both	Mesh	6	27	12	AAS, NRS
Eklund [34]	2010	RCT	269	22	Both	Mesh	3^s^	16^s^	5^s^	Ad-hoc, IPQ^s^
Reinpold [35]	2011	Observational	127	12	Open	Both	6^t^	17	1	VAS

AAS, activities assessment scale; Ad-hoc: ad-hoc questionnaire; DIBS: duration-intensity-behavior scale; IPQ, inguinal pain questionnaire; MPQ, McGill pain questionnaire; MRC LGH, medical research council laparoscopic groin hernia trial group; NRS, numeric rating scale; RCT, randomised controlled trial; SF-36, short-form 36; TAPP, transabdominal preperitoneal repair; TEP, total extraperitoneal repair; UCSF, University of California and San Francisco Pain Service patient questionnaire; VAS, visual analogue scale; VRS, verbal rating scale; -, No applicable data were reported or reported data were contradictory.

^a^ Defined as either a review, an RCT, or an observational study.

^b^ Google Scholar searched on 23 May 2022.

^c^ Defined as either pain interfering with activities of daily living or pain of at least moderate intensity.

^d^ Follow-up at 12 months.

^e^ 29% of patients receiving laparoscopic repair reported pain, and 37% of patients receiving open repair reported pain.

^f^ Questionnaire included cherry-picked items from the MPQ, SF-36, and UCSF.

^g^ At 3 months, a modified version of SF-36 was applied with added ad-hoc items. At later follow-up, the Wisconsin Brief Pain Questionnaire was used. Severe or very severe pain was reported by 3%, but the applied scale did not include a “moderate” response option.

^h^ Pain and discomfort was reported collectively, median follow-up was 21 months.

^i^ This review found frequencies of pain of up to 54% in the included studies. Studies with minimum 3 months of follow-up were included in the review. Median follow-up in the included studies ranged from 3 to 72 months.

^j^ Minimum 6 months of follow-up was an inclusion criterion, but mean follow-up ranged from 26 to 31 months between groups.

^k^ Follow-up performed at 12, 24, 36, and 60 months. At 12 months, 28% of patients receiving laparoscopy and 36% of patients receiving open repair reported pain. The indicated 32% is an approximation of the overall rate, and 3% is an approximation of the overall rate of severe or very severe pain. The applied scale did not include a “moderate” response option.

^l^ 15% of patients receiving TAPP, 31% of patients receiving Lictenstein, and 36% of patients receiving Shouldice reported any pain at a median follow-up of 52 months. Moderate to severe pain was reported by 1%, 9%, and 16%, respectively. The indicated 29% and 9% are approximations of the overall rates.

^m^ Studies with minimum 6 months follow-up were included in the review.

^n^ Follow-up at 1, 3, and 12 months, but only the last was characterised as chronic pain, and 40% of patients who received lightweight mesh and 52% who received heavyweight mesh reported any pain at 12 months, respectively. Severe to very severe pain was reported by 3%–4% at 12 months. Data on moderate pain was not extractable. The presented 42% and 3% are approximations of the overall rates. Questionnaire included VAS and a modified SF-36.

^o^ Purports to adhere to the definition by the International Association for the Study of Pain (i.e., 3 months) but only data from 6 months follow-up is reported.

^p^ The range of follow-up was 24–36 months.

^q^ Median follow-up in the included studies was 21 months, and the frequency of significant pain could not be reproduced.

^r^ Follow-up ranged from 6 to 84 months.

^s^ Chronic pain was defined as pain beyond 3 months, but the earliest follow-up was at 12 months. 11% of patients receiving TEP, and 22% of patients receiving Lichtenstein reported pain at 12 months. The presented 16% and 5% are approximations of the overall rates. Pain degree was further characterised using the IPQ.

^t^ Follow-up ranged from 6 to 9 months.

Five of the included studies clearly defined chronic pain to be pain that persisted at least 3 months after surgery [20, 21, 23, 31, 34], which is in accordance with the original definition of chronic pain by the International Association for the Study of Pain (IASP) [36]. Four studies clearly defined chronic pain as pain persisting beyond 6 months postoperatively [24, 27, 33, 35], and the remaining studies did not clearly define the timeframe of chronic pain other than pain present at long-term follow-up.

The reported rates of chronic pain of any degree in the studies ranged from 10% to 63%, and this outcome was reported by all studies (inclusion criterion). The rate of a moderate-to-severe degree of chronic pain or pain that interfered with activities of daily living ranged from 1% to 18%, and this outcome was reported in the majority of the studies [16, 17, 19, 21, 22, 25, 26, 28–30, 32–35]. In the included studies published between 1996 and 2004, the reported rates of chronic pain of any degree ranged from 19% to 63%, and for the studies published between 2005 and 2011 these numbers were 10%–42%. In the studies defining chronic pain as persistent pain beyond 3 months, the rates ranged from 16%–54%, and in the studies using a 6-month definition, the rates ranged from 10%–23%.

Many different means of measurement were used. Ten of the studies used either an unvalidated ad-hoc questionnaire or a heavily modified version of a previously validated questionnaire [17–22, 24, 25, 28, 34]. Five studies used either a verbal rating scale or a visual analogue scale as their primary means for measuring pain, however, some were supplemented with ad-hoc questions about pain impact [16, 26, 28, 29, 35].

Another important aspect is the timespan patients were surveyed about in each study. In five of the included studies, patients were asked about any pain in the past week [18, 21, 25, 32, 34], three other studies asked about any pain experienced within the past month [17, 19, 24], and yet another study asked about pain in the past 2 weeks [35]. One study assessed both the level of current pain, the worst pain experienced in the past week, and the frequency of pain during the past week [30]. In another study, patients were retrospectively asked if they recalled experiencing pain during the first 3 months postoperatively, but this was assessed at a follow-up ranging from 21 to 57 months [20]. The remaining six studies (not considering the three included systematic reviews) either specified that only current pain was measured or did not explicitly qualify the timeframe [16, 22, 26, 28, 29, 33].

The included publications were published between 1996 and 2011, thus predating the wider implementation of laparoscopic repair [37]. Accordingly, several of the studies included only open repairs [16, 17, 20, 24, 28, 29, 35] or mainly open repairs [19, 21, 30, 32].

Two of the systematic reviews performed meta-analyses and provided pooled estimates of chronic pain, which were 12% and 11%, respectively [27, 31]. However, both estimates are subject to considerable uncertainty due to large methodological heterogeneity, and neither of the reviews provided any measures of statistical heterogeneity. The third included systematic review did not perform a meta-analysis and did not calculate a pooled estimate of the rate of chronic pain, but only provided a range from 0% to 53% [23].

### Risk of Bias Assessment

We assessed the included original studies with the JBI, and the results are presented in Table 2. Only one study was given a perfect score [30], and the remaining studies presented various methodological issues limiting the external validity of their reported chronic pain rates. We evaluated the three included systematic reviews using the AMSTAR2, which resulted in a grading of “critically low quality” for all three [23, 27, 31]. Detailed results are available in Table 3.

**TABLE 2:** Risk of bias assessment of included original studies using the JBI.

Checklist item	Cunningham [16]	Callesen [17]	MRC LGH Trial Group [18]	Bay-Nielsen [19]	Poobalan [20]	Courtney [21]	Kumar [22]	Bay-Nielsen [24]	Grant [25]	Köninger [26]	O´Dwyer [28]	Alfieri [29]	Fränneby [30]	Kalliomäki [32]	Aasvang [33]	Eklund [34]	Reinpold [35]
1) Was the sample representative of the target population?	Unclear	Yes	Yes	Yes	Yes	Yes	Yes	Unclear	Yes	Yes	Yes	Yes	Yes	Yes	Yes	Unclear	Unclear
2) Were study participants recruited in an appropriate way?	Yes	No	Yes	Yes	No	Yes	No	Yes	Yes	No	Yes	Yes	Yes	Yes	Yes	Yes	No
3) Was the sample size adequate?	No	No	Yes	Yes	No	Yes	No	Yes	Yes	No	No	Yes	Yes	Yes	No	Yes	No
4) Were the study subjects and setting described in detail?	No	Yes	Yes	Yes	Yes	Yes	Yes	No	No	Yes	Yes	Yes	Yes	Yes	Yes	No	No
5) Is the data analysis conducted with sufficient coverage of the identified sample?	No	Yes	Yes	Yes	Yes	Yes	Yes	Yes	Yes	No	Yes	Yes	Yes	Yes	Yes	Yes	Yes
6) Were objective, standard criteria used for measurement of the condition?	No	No	No	No	No	No	No	No	No	No	No	No	Yes	Yes	Yes	No	No
7) Was the condition measured reliably?	No	Yes	No	Yes	Yes	Yes	Yes	Yes	Yes	No	No	No	Yes	Yes	Unclear	No	No
8) Was there appropriate statistical analysis?	No	Yes	No	No	No	No	No	No	No	No	No	No	Yes	No	No	No	No

JBI, Joanna Briggs Institute Checklist for Prevalence Studies. Details on each checklist item are available in the literature [14].

**TABLE 3:** Systematic critical appraisal of included systematic reviews using the AMSTAR2.

Checklist item	Poobalan [23]	Aasvang [27]	Nienhuijs [31]
1) Did the research questions and inclusion criteria for the review include the components of PICO?	Yes	Yes	Yes
2) Did the report of the review contain an explicit statement that the review methods were established prior to the conduct of the review and did the report justify any significant deviations from the protocol?	No	No	No
3) Did the review authors explain their selection of the study designs for inclusion in the review?	Yes	No	No
4) Did the review authors use a comprehensive literature search strategy?	Partial yes	Partial yes	Yes
5) Did the review authors perform study selection in duplicate?	No	No	Yes
6) Did the review authors perform data extraction in duplicate?	No	No	No
7) Did the review authors provide a list of excluded studies and justify the exclusions?	No	No	No
8) Did the review authors describe the included studies in adequate detail?	Partial yes	No	No
9) Did the review authors use a satisfactory technique for assessing the risk of bias (RoB) in individual studies that were included in the review?	No	No	No
10) Did the review authors report on the sources of funding for the studies included in the review?	No	No	No
11) If meta-analysis was performed did the review authors use appropriate methods for statistical combination of results?	—	No	Yes
12) If meta-analysis was performed, did the review authors assess the potential impact of RoB in individual studies on the results of the meta-analysis or other evidence synthesis?	—	No	No
13) Did the review authors account for RoB in individual studies when interpreting/discussing the results of the review?	No	No	No
14) Did the review authors provide a satisfactory explanation for, and discussion of, any heterogeneity observed in the results of the review?	Yes	Yes	Yes
15) If they performed quantitative synthesis did the review authors carry out an adequate investigation of publication bias (small study bias) and discuss its likely impact on the results of the review?	—	No	No
16) Did the review authors report any potential sources of conflict of interest, including any funding they received for conducting the review?	Yes	Yes	No
Overall grade	Critically low quality	Critically low quality	Critically low quality

AMSTAR2, A Measurement Tool to Assess systematic Reviews 2; PICO, population, intervention, comparator, outcome; RoB, risk of bias. Details on each checklist item are available in the literature [15].

## Discussion

In this review, we wanted to demonstrate the uncertainty that remains about the rate of chronic pain after inguinal hernia repair. This uncertainty is partly due to the heterogeneity in the definition and measurement of chronic pain as well as the recent advancements in modern surgery that may have resulted in a decreasing chronic pain rate, which is not yet fully reflected in the literature. This illustrates that more contemporary research on the topic of chronic pain is clearly warranted.

These possibly outdated chronic pain rates continue to be repeated in the literature and are routinely communicated to patients preoperatively. We want to urge hernia researchers to refrain from repeating this potentially outdated information in future publications. Newer sources with more contemporary and higher quality evidence do already exist, and some of these have reported chronic pain rates as low as 3% for laparoscopic repair [38, 39]. These and other recent sources should be given focus going forward.

An agreed definition of chronic pain and more uniformity in its assessment is necessary. We do not propose a specific definition here, but we want to emphasise the critical necessity of a uniform definition based on international consensus, preferably with patient involvement, as well as agreed and standardised methods of measurement. This is not a novel notion, but it is still as important and relevant as ever [8, 9], and hernia researchers as well as international hernia societies must address and remedy this issue. Consequently, we must conduct new, well-designed, prospective multicentre studies based on modern surgical technique and quality to establish a more accurate and contemporary chronic pain rate—a rate that reflects the recent advances in surgical quality and that eventually will benefit patients through better hernia research. Furthermore, the existing hernia registries, as well as the several upcoming registries, provide large datasets of increasing quality and with high external validity. Going forward, large registry-based studies are likely to produce some of the most accurate estimates of the rate of chronic postoperative inguinal pain. The effects of specific patient characteristics, of surgeons’ expertise, and of the chosen surgical technique on contemporary chronic pain rates need to be addressed thoroughly in future studies.

### Temporal Definition of Chronic Pain

A major reason for the heterogeneity and inaccuracy in the reported chronic pain rates is that the temporal aspect is disputed. Chronic pain is most commonly defined as pain persisting either three or 6 months postoperatively [9], but a 1-year threshold has also been proposed [8]. The international treatment guidelines have not yet agreed on this [5, 6]. A 3-month threshold is in line with the original IASP definition of chronic pain [36] and the recommendations by the HerniaSurge Group [5], but some argue that 6 months are necessary after mesh-based hernia repairs to allow for the mesh-related inflammatory response to decrease [4]. The included studies using a 3-month threshold reported chronic pain rates ranging from 16%–54%, and the studies using af 6-month threshold reported rates ranging from 10%–23%. In hernia surgery, a 6-month threshold may be a more accurate reflection of the pathophysiological transition from acute to chronic pain, even though the exact mechanism behind this transition is not entirely clear [4–6, 27]. The aetiology of chronic pain after inguinal hernia repair is likely to be multifactorial, but it is mostly thought to be of neuropathic origin, which justifies the extensive attention given to intraoperative nerve management [5].

The ICD-11 definition [40] and the current IASP definition specific for chronic postsurgical pain [41] maintain that chronic pain is pain persisting 3 months postoperatively. This threshold is standardised across all surgical procedures, but seemingly for pragmatic purposes rather than pathophysiological [40], and these definitions do not seem to explicitly consider the potential influence of implants, such as meshes. Most important, however, is that an operational definition for this is determined through scientific consensus.

In 2020, IASP updated its general definition of pain [42]. The new definition maintains an emphasis on pain as a personal subjective experience, but it also slightly downplays the role of tissue damage as the source of pain. However, this is unlikely to have direct implications for the definition of chronic pain, but we are not aware of any reports or responses from the hernia research community on this yet.

In the included studies, there was a large variation in the length of follow-up (total range of 3–84 months). However, it is important to note that postoperative pain declines substantially over time [38], and with a distinct decrease at around 3.5 years postoperatively for laparoscopic repairs [43]. This may account for some of the reported variation, and many of the chronic pain rates reported in the included publications are in fact incomparable because of the temporal decline in pain.

### Severity of Chronic Pain

Many different rates of chronic pain have been disseminated throughout the literature and have been reproduced in hundreds of papers, often with reference to the publications included in this review (Table 1). However, most of these rates are based on reports of pain of any severity, i.e., “any pain”, which arguably is a substantial overestimation of what matters to the patients, and that is presumably a level of pain that interferes with their daily activities or their quality of life. These concepts are more complicated to assess accurately, and they require validated multi-dimensional tools. They are likely to be more meaningful and consequential for patients, however, firm evidence of patient preferences on these issues is still lacking, and it is a topic that surely needs further research.

Two of the included clinical trials applied “any pain” as a primary endpoint, but none applied moderate-to-severe pain as primary endpoint, which may be criticised [18, 28]. Even though the rate of moderate-to-severe pain is a rarer event requiring a greater sample of patients to achieve a high enough power, it is likely a more important and meaningful endpoint for the patients [44, 45]. A sufficient sample size can be achieved by multicentre or international collaboration.

The majority of the studies selected for this review [16–20, 22–24, 26, 28, 30, 31, 34, 35] reported exceptionally high rates of chronic pain in their respective abstracts, where most of them were based on the “any pain”-definition. Many of the publications also reported rates of significant/severe pain with lower associated rates, however, the higher rates of “any pain” reported in abstracts tend to be reproduced in the literature. It could be speculated that a fear of publication bias might have influenced this, and this may contribute to an inflated perception of the extent of the problem of chronic pain.

### Measurement of Chronic Pain

The measurement of pain is by definition indirect and complex [46], and most of the included studies suffer from issues regarding the measurement of pain. A factor contributing to the variation of reported chronic pain rates is the abundance of different methods of measurement available, of which many are suboptimal [47]. Some studies use unidimensional tools, such as a verbal rating scale or a visual analogue scale, which may be excellent for measuring acute pain but are generally not considered sufficient measures for the complex nature of chronic pain [46, 48]. Unvalidated and unidimensional instruments are an unreliable approach, and validated multi-dimensional measures are necessary [46].

Another important aspect is the timing of measurements: when is pain measured and which timespan are patients asked to account for. Assessing a patient’s current level of pain is only a snapshot, which is susceptible to random interference from unrelated factors without reflecting the natural day-to-day fluctuations [46]. Alternatively, patients could be asked to assess pain experienced during a preceding period of a specified length (days/weeks/months), which is probably a more stable measure, but it does entail a risk of recall bias. Retrospective assessment of previously experienced pain is even more at risk of recall bias, as pain memory is notoriously unreliable, and it is dependent on numerous contextual factors including a patient’s current experience of pain [46, 49]. The preferable timing of measurements remains a topic for further discussion, however, prospective measurements should be favoured [50].

A highly speculative aspect of pain measurement is that patients might overexpress their experience of pain as a result of being surveyed about it. This is known as a negative Hawthorne effect and might account for a small portion of the high rates of chronic pain reported in the literature [51].

### Advances in Inguinal Hernia Surgery

In this review, we have selected the most cited and impactful publications reporting on the rate of chronic pain. Evidently, most highly cited studies are older, and the most recent study included here was published in 2011 [35]. However, all of the included studies remain frequently cited to this day, which is remarkable considering the rapid developments in the quality of hernia surgery in recent years [12]. A majority of the included studies involved only or predominantly open repairs, but these publications do not reflect current surgical practice in many developed countries, as laparoscopy has become increasingly popular [37] and is associated with a lower risk of chronic pain [38, 52, 53]. As described, several factors are contributing to higher quality in surgical practice, which hopefully have already translated into lower chronic pain rates. With all of this in mind, the conventionally reported rates of chronic pain are likely outdated.

### Clinical Implications

Currently reported chronic pain rates likely overestimate the actual rate in 2022 or at least the rate of pain of a degree that is meaningful to patients. These seemingly excessive rates are widely reported in the literature, they are easily available to patients online [54], and patients are routinely informed about them during preoperative visits. Presumably, this could deter patients from receiving necessary surgery if they are scared off by potentially obsolete and misleading information.

In general, an accurate estimate of the extent of a problem is necessary to determine proportionate preventive measures and decide appropriate therapeutic efforts [7]. This is certainly also true in the case of chronic pain after inguinal hernia surgery. A more accurate and contemporary estimation of the chronic pain rate is critically necessary to better inform shared decision-making between patients and surgeons based on current and high-quality evidence. Ultimately, patients deserve an accurate basis for an informed choice about their own treatment.

In addition to an overall chronic pain rate, it might also be informative and valuable for patients to assess and communicate demographic-specific rates whenever possible. The risk of chronic pain is dependent on multiple different demographic variables, and predictive modelling and individualised risk estimation partially based on these may be a beneficial approach going forward [55].

### Limitations

This study is an informal review of 20 high-impact studies. It is not a comprehensive systematic review of the entire literature, and due to the nature of this study, it does not include more contemporary research. For this, we refer to the literature [38, 39].

The applied search engine, Google Scholar, is an excellent source of freely available bibliometric data, however, it does provide some limitations. In general, Google Scholar identifies more citations than comparable search engines, and it includes more non-journal citations (i.e., books, conference abstracts, grey literature, etc.) [56], which could lead to a risk of overestimating the actual impact of the included studies. Google Scholar utilises an effective but also non-transparent search algorithm, meaning that literature searches have poor reproducibility, which is a limitation of this search engine [56, 57]. Google Scholar only allows for access to the 1,000 most relevant search results for any particular search query, and thus, the literature search in this review cannot be considered entirely comprehensive [58]. However, the search algorithm determines the relevance of search results partially based on citation count, which effectively minimises the risk of missing any highly cited records [59].

In this review, the inclusion of studies was partially based on the overall citation count of each study. This was a pragmatic approach, and the number of citations is only a surrogate measure of study impact or quality, and the reliability and accuracy of this measure is debatable. Inclusion was also based on number of citations per year since publication, which was an ad-hoc measure implemented to ensure contemporary relevance of the included studies and to avoid including no longer cited studies. However, both of these inclusion criteria discriminate against newer publications. This study was limited by the fact that the applied thresholds (>100 overall citations, >10 citations per year) were entirely arbitrary. Furthermore, this review may be vulnerable to selection bias, as the included highly cited studies may have been more prone to some of the methodological pitfalls described above than studies with lower citation counts.

We selected the JBI for the risk of bias assessment in this review, because it is a tailored tool for studies reporting prevalence [14]. However, it should be noted that many such tools exist [60, 61], and it has previously been suggested that the JBI may not be an optimal tool, but it was included in the present study as it is likely the best available tool for this purpose [60, 61]. The JBI is designed exclusively for the evaluation of prevalence data, and the results produced by the JBI should not be interpreted otherwise. The poor evaluation of most of the included studies (Table 2) is primarily a reflection of the fact that most of these studies were likely never intended to produce generalisable estimates of prevalence or incidence. Accordingly, the presented results indicate nothing about other aspects of the studies. An additional limitation of the JBI is that it does not provide an overall score for each evaluated study, which can make it difficult to operationalise its results in a systematic review or meta-analysis.

For the reasons above, we do not claim this study to be exhaustive. Nevertheless, the results and conclusions remain valid for the included studies.

### Conclusion

In this review, we have explained and demonstrated that the chronic pain rates conventionally reported after inguinal hernia repair in the literature are obsolete, probably inaccurate, and likely exaggerated. This is due to uncertainties about the definition and measurement of chronic pain, other methodological shortcomings, and the fact that recent advances in inguinal hernia surgery are not reflected in the included publications. We have also highlighted the importance of solving these issues by determining consensus-based definitions and standards, and subsequently performing large, well-designed studies to establish a more accurate chronic pain rate. For this, we need prospective multicentre studies that apply clear evidence- and consensus-based definitions, use validated measurement instruments, and are reflective of current surgical practice and quality.
