# Supplementary material for: A Critical Appraisal of the Chronic Pain Rate After Inguinal Hernia Repair
Source: J Abdom Wall Surg. 2023 Jan 19;2:10972. doi: 10.3389/jaws.2023.10972 (PMC10831660; doi:10.3389/jaws.2023.10972)
Supplement: Supplementary file 1 [file DataSheet1.DOC]

**Supplementary Data Sheet 1** – illustration of experimental protocols.

Example Protocol A (used for 7 subjects):

| **Run 1** | F1-T0-B | F1-T1-B | F1-T2-PG | F1-T3-PG | F2-T3-WB | F2-T2-WB | F2-T1-G | F2-T0-G |
| --- | --- | --- | --- | --- | --- | --- | --- | --- |
| **Run 2** | F2-T1-B | F2-T3-PG | F2-T0-B | F2-T2-PG | F1-T2-WB | F1-T0-G | F1-T3-WB | F1-T1-G |
| **Run 3** | F2-T2-WB | F2-T0-G | F2-T3-WB | F2-T1-G | F1-T1-B | F1-T3-PG | F1-T0-B | F1-T2-PG |
| **Run 4** | F1-T3-WB | F1-T2-WB | F1-T1-G | F1-T0-G | F2-T0-B | F2-T1-B | F2-T2-PG | F2-T3-PG |
| **Run 5** | F2-T3-PG | F2-T2-PG | F2-T1-B | F2-T0-B | F1-T0-G | F1-T1-G | F1-T2-WB | F1-T3-WB |
| **Run 6** | F1-T2-PG | F1-T0-B | F1-T3-PG | F1-T1-B | F2-T1-G | F2-T3-WB | F2-T0-G | F2-T2-WB |
| **Run 7** | F1-T1-G | F1-T3-WB | F1-T0-G | F1-T2-WB | F2-T2-PG | F2-T0-B | F2-T3-PG | F2-T1-B |
| **Run 8** | F2-T0-G | F2-T1-G | F2-T2-WB | F2-T3-WB | F1-T3-PG | F1-T2-PG | F1-T1-B | F1-T0-B |

Example Protocol B (used for 7 subjects):

| **Run 1** | F2-T0-B | F2-T1-B | F2-T2-PG | F2-T3-PG | F1-T3-WB | F1-T2-WB | F1-T1-G | F1-T0-G |
| --- | --- | --- | --- | --- | --- | --- | --- | --- |
| **Run 2** | F1-T1-B | F1-T3-PG | F1-T0-B | F1-T2-PG | F2-T2-WB | F2-T0-G | F2-T3-WB | F2-T1-G |
| **Run 3** | F1-T2-WB | F1-T0-G | F1-T3-WB | F1-T1-G | F2-T1-B | F2-T3-PG | F2-T0-B | F2-T2-PG |
| **Run 4** | F2-T3-WB | F2-T2-WB | F2-T1-G | F2-T0-G | F1-T0-B | F1-T1-B | F1-T2-PG | F1-T3-PG |
| **Run 5** | F1-T3-PG | F1-T2-PG | F1-T1-B | F1-T0-B | F2-T0-G | F2-T1-G | F2-T2-WB | F2-T3-WB |
| **Run 6** | F2-T2-PG | F2-T0-B | F2-T3-PG | F2-T1-B | F1-T1-G | F1-T3-WB | F1-T0-G | F1-T2-WB |
| **Run 7** | F2-T1-G | F2-T3-WB | F2-T0-G | F2-T2-WB | F1-T2-PG | F1-T0-B | F1-T3-PG | F1-T1-B |
| **Run 8** | F1-T0-G | F1-T1-G | F1-T2-WB | F1-T3-WB | F2-T3-PG | F2-T2-PG | F2-T1-B | F2-T0-B |

Condition coding:

**Visual Field:**

F1 – Right Visual Field

F2 – Left Visual Field

**Task and Visual Feedback:**

T0 – Target-Directed / visual feedback

T1 – Target-Directed / no visual feedback

T2 – Allocentric / visual feedback

T3 – Allocentric / no visual feedback

**Instruction:**

G - Green

B - Black

PG - Pink to Green

WB - White to Black

NOTE: the order of instructions (G, B, GB, WB) varied randomly from subject to subject. Thus, an equivalent protocol would be obtained by exchanging ‘Green’ with ‘Black’ and ‘Pink to Green’ with ‘White to Black’, respectively.
