# Supplementary material for: A Critical Appraisal of the Chronic Pain Rate After Inguinal Hernia Repair
Source: J Abdom Wall Surg. 2023 Jan 19;2:10972. doi: 10.3389/jaws.2023.10972 (PMC10831660; doi:10.3389/jaws.2023.10972)
Supplement: Supplementary file 2 [file DataSheet2.DOC]

Supplementary Data Sheet 2 – Results of 2x2x2 ANOVA analyses, with ‘task, ‘visual field’ and ‘visual feedback’ as repeated measures factors. Stereotactic coordinates (Talairach and Tournoux, 1988) and F-values represent the average value across voxels. *BF:* Detected using Bonferroni corrected significance thresholds (p<.1). *CLU* – Detected using cluster size thresholds (p<.0001). We first evaluated the ANOVA results using BF thresholds. When no significant result was found in an area previously identified using conjunction analyses (see Table 1 and 3, main text), we subsequently used CLU thresholds to evaluate ANOVA results. CLU is less conservative than BF. Thus, by using both BF and CLU , we were able to determine if the ‘lack of activity’ in the ANOVA as compared to our conjunction analysis was due to the more conservative statistical criterion as introduced by BF, or due to a qualitatively different result. For more details see main text.

| Label | # voxels (1 mm^3^) | Tal x | Tal y | Tal z | Avg. F(1,13) | Type-I error correction |
| --- | --- | --- | --- | --- | --- | --- |
| **Main Effect of Visual Feedback** | | | | | |  |
| IPS(posterior)(L) | 785 | -24 | -59 | 48 | 94.718 | BF |
| IPS(posterior)(R) | 402 | 26 | -58 | 50 | 80.195 | BF |
| SPOC(L) | 2705 | -19 | -84 | 25 | 96.171 | BF |
| SPOC(R) | 1352 | 19 | -83 | 31 | 89.223 | BF |
| MT+(L) | 3909 | -41 | -72 | -1 | 99.409 | BF |
| MT+(R) | 4983 | 41 | -68 | 2 | 119.348 | BF |
| **Main Effect of Task** | | | | | |  |
| PMd(L) | 1144 | -23 | -9 | 52 | 37.426 | CLU |
| PMd(R) | 947 | 24 | -8 | 54 | 35.176 | CLU |
| IPS(Fundus) (L) | 558 | -44 | -43 | 42 | 82.370 | BF |
| IPS(posterior)(R) | 10 | 21 | -66 | 42 | 70.072 | BF |
| IPS/SPL(posterior)(L) | 16 | -19 | -69 | 52 | 69.994 | BF |
| **Interaction between Task and Visual Field** | | | | | |  |
| LO (L) | 197 | -39 | -75 | -5 | 32.999 | CLU |
| LO (R) | 52 | 43 | -72 | -9 | 32.195 | CLU |
| FFG (R) | 118 | 28 | -70 | -15 | 33.440 | CLU |
| **Interaction between Task and Visual Feedback** | | | | | |  |
| PreSMA (R) | 30 | 6 | 9 | 48 | 31.710 | CLU |
| PMd(L) | 475 | -24 | -7 | 55 | 33.685 | CLU |
| PMd(R) | 57 | 20 | -4 | 47 | 31.685 | CLU |
| IPS (L) | 734 | -43 | -44 | 38 | 39.907 | CLU |
| IPS / SPL (L) | 502 | -14 | -73 | 51 | 34.560 | CLU |
| IPS (posterior) (L) | 190 | -28 | -65 | 39 | 32.767 | CLU |
| PPC / OCC (L) | 71 | -29 | -76 | 21 | 32.315 | CLU |
| IPS (R) | 1019 | 46 | -43 | 46 | 33.977 | CLU |
| IPS / SPL (R) | 283 | 17 | -77 | 46 | 33.013 | CLU |
| PPC/OCC(R) | 739 | 33 | -78 | 25 | 37.339 | CLU |
